# Massachusetts Veterinary Medical Association

**Published:** 1892-04

**Authors:** Austin Peters

**Affiliations:** Secretary


*Massachusetts Veterinary Medical Association.*—The regular meeting of the Massachusetts Veterinary Association was held at 19 Boylston Place, Boston, Wednesday evening, February 24th, at 7.30 o'clock; President L. H. Howard in the chair. Members present: Drs. Bryden, Blackwood, Bunker, Becket, Emerson, Hadcock, Howard, Osgood. Winchester, Winslow, Parker, and the Secretary; honorary member, Dr. Stickney. Visitors: Drs. W. L. LaBaw and F. B. Carlton; also Messrs. G. Sawyer, Geo. Quinlan and Murch, senior students at the Harvard Veterinary School.

The records of the last meeting read and accepted. Unfinished business: Dr. Winchester reported the result of the meeting of the Comitia Minora of the U. S. V. M. A. in New York recently; that it was decided to hold the annual meeting of the Association in Boston, upon the third Tuesday, Wednesday and Thursday in September. Motion made by Dr. Blackwood, and seconded by Dr. Bunker, that the report be accepted, and a vote of thanks be extended the committee. Carried. Reports of Committees: Dr. Bryden, Chairman of the Committee, to wrtie to the Governor regarding the Cattle Commission, reported for his Committee. Motion made by Dr. Winchester and seconded by Dr. Blackwood that the report be accepted, and a vote of thanks be given the committee. Carried. Motion made by Dr. Bunker, seconded by Dr. Blackwood, that the committee report be put upon the records of this Association. Carried. The report is as follows:

"BOSTON, MASS., January 27th, 1892.

"*To His Excellency,*
"WILLIAM E. RUSSELL,
"*Governor of the State of Massachusetts.*

"SIR:

"We, a Committee appointed by the Massachusetts Veterinary Association for the purpose, January 27, 1892, desire to represent to Your Excellency that in the opinion of the representative body of the profession in this State, that any important question relating to the contagious diseases of animals should properly be referred in its finality to a member, or members, of our profession for decision.

"It desires to make no suggestion as to how this result shall be accomplished, but simply to state that in its opinion all questions relating to contagious diseases of animals bear the same relation to the veterinary profession that contagious diseases of the human subject bear to the medical profession.

(Signed) "WILLIAM BRYDEN, V. S.
" "FREDERICK H. OSGOOD, M. R. C. V. S.
" "LESTER H. HOWARD, D. V. S.,
*Committee.*"

The following was received in reply:

"*Commonwealth of Massachusetts,*
"*Executive Department,*
"BOSTON, January 29th, 1892.

"MR. WILLIAMSON BRYDEN,
"36 Sudbury Street, Boston, Mass.

"MY DEAR SIR:

"I have received the opinion of your Committee submitting the action of the Massachusetts Veterinary Association in reference to the necessity of having skilled and professional opinion upon questions relating to contagious diseases of animals.

"I fully agree with the views of your Association, and shall endeavor, as far as is in my power, to carry out those views.

"Very truly yours,
(Signed.) "WM. E. RUSSELL."

Admission of members: Name of John B. Paige, D. V. S. (Mont.) Professor of Veterinary Science at the Massachusetts Agricultural College, Amherst, was voted upon. Twelve ballots were cast, all in the affirmative, and Dr. Paige was accordingly elected. Applications for membership were received from Drs. W. L. LaBaw and F. B. Carlton. New business: Dr. Winchester proposes that we take some action now towards arranging a program for the meeting of the U. S. V. M. A. here next September, and thinks a Committee of Arrangements should at once be appointed to make hotel and railroad rates. Dr. Bunker approves Dr. Winchester's remarks and files the following motion: "That a committee of five be appointed, including the President and Secretary of this Association, to find out and report at our next meeting what arrangements can be made for the entertainment of the U. S. Association as regards hotels, railroads, and all other matters relating to the same." Seconded by Dr. Osgood. Dr. Bryden agrees with Dr. Winchester's ideas. Motion carried. The Chair appointed Drs. Winchester, Bunker and J. S. Saunders on the committee, with the President and Secretary members ex-officio.

Dr. Bryden then read a paper upon "Navicular Disease," after which a discussion followed, taken part in by the members present.

Dr. Osgood, who has recently been appointed Professor of Veterinary Surgery at the Harvard Veterinary School, announces that he has taken *entire charge* of the Harvard Veterinary Hospital, and is ready to extend any courtesy he can to this Association, and will be happy to receive the co-operation of its members.

Dr. Bunker promised a paper for the March meeting. Motion made by Dr. Bunker, seconded by Dr. Blackwood, that a vote of thanks be given Dr. Bryden for his paper. Carried. Motion was made by Dr. Winchester, seconded by Dr. Emerson, that hereafter the senior students at the Harvard Veterinary School be invited to the meetings of this Association. Carried. Meeting then adjourned.

AUSTIN PETERS, *Secretary.*

